# Immunomodulatory Effects of *Cistanche deserticola* Polysaccharides on LPS-Induced Immune Damage in RAW264.7 Macrophages with Transcriptome Analysis

**DOI:** 10.3390/antiox15101236

**Published:** 2026-09-24

**Authors:** Baotang Zhao, Faqin Tao, Shengfang Wang, Jing He, Shen Song

**Affiliations:** 1 College of Food Science and Engineering, Gansu Agricultural University, Lanzhou 730070, China; 2 Lanzhou Vocational Technical College, Lanzhou 730070, China; faqintao@126.com; 3 College of Life Science, Northwest Normal University, Lanzhou 730070, China

**Keywords:** *Cistanche deserticola* polysaccharides, immunomodulation, transcriptome

## Abstract

Polysaccharides (CDP-D2-N1) were extracted from *Cistanche deserticola* and used as an immunomodulatory substance to control inflammation and cytokines. This article chose LPS-induced inflammation model in RAW264.7 as the study subject and discloses the immune-inflammatory responses of CDP-D2-N1for lipopolysaccharide (LPS)-stimulated RAW264.7 cells. We investigated the screening of immunomodulatory activity of CDP-D2-N1 on LPS-induced RAW264.7 and the study of its pathway of action. CCK-8, qPCR and other tests were used to screen the CDP-D2-N1 with repairing effects on RAW264.7. The results show that CDP-D2-N1 pretreatment for 12 h at a concentration of 62.5–250 μg/mL improved the phagocytosis of RAW264.7, activated the expression of the TLR4 receptor, inhibited the expression of proinflammatory factors (TNF-α, IL-1, and IL-6) and the creation of iNOS, and modulated the NO of cell signaling molecules in RAW264.7 cells, thus improving the immunity of RAW264.7 cells and the activity of immune injury RAW264.7 cells. The combination of transcriptomics and p38 inhibitor analysis showed that CDP-D2-N1 could attenuate LPS-induced RAW264.7 cells’ immune injury by affecting the regulation of MAP3/6 expression in the TNF signaling pathway.

## 1. Introduction

*Cistanche deserticola* (Orobanchaceae), commonly known as desert living cistanche, a traditional edible food, was officially approved as a medicinal and food homologous material in November 2023 in China. Accumulating evidence indicates that *Cistanche deserticola* polysaccharide (CDP) exerts therapeutic effects against various pathologies, including foot-and-mouth disease, ischemic stroke, osteoporosis, depression, and slow transit constipation, while also providing neuroprotection and immunostimulatory benefits [1,2,3,4,5].

Inflammation is a fundamental physiological and immune response to cellular and tissue injury, typically triggered by pathogen invasion, including viruses and bacteria. The subsequent release of reactive oxygen species (ROS), reactive nitrogen species (RNS), and pro-inflammatory mediators initiates the organism’s restorative processes. *Cistanche deserticola* polysaccharide (CDP) has demonstrated potent anti-inflammatory effects across various in vitro and in vivo models, mitigating damage induced by lipopolysaccharide (LPS), alcohol, halothane, oxygen–glucose deprivation/reperfusion, gentamicin [1,6,7,8,9], and surgical interventions like ovariectomy [10,11]. These biological activities are attributed to reductions in malondialdehyde (MDA), the elevation of glutathione (GSH) and superoxide dismutase (SOD) levels, the preservation of mitochondrial function, the activation of the Nrf2/Keap1 signaling pathway, and the scavenging of free radicals [1,6,7,8,9,10,11]. Furthermore, the bioactivity of CDP is highly dependent on its structural characteristics, specifically monosaccharide composition and molecular weight. For instance, an arabinogalactan fraction rich in Gal and Glc from *Cistanche deserticola* stems has been shown to stimulate lymphocyte proliferation [12]. Similarly, a CDP fraction comprising 81.2% Glc, 3.7% Rha, 3.1% Ara, 7.1% Gal, and 4.9% Gal-UA significantly ameliorated constipation symptoms [5]. Wang et al. reported the hepatoprotective effects of CDP against liver inflammation [9], while recent studies employing microbiome and metabolome analyses have elucidated its antidepressant-like effects [4]. Although transcriptomic and proteomic profiling have been widely applied to elucidate the antiviral, immunomodulatory, antidiabetic, and anti-inflammatory functions of various polysaccharides [13,14,15,16], specific multi-omics data regarding the anti-inflammatory mechanism of CDP remain lacking. Analogous studies on *Isatis indigotica* polysaccharides have demonstrated that integrated transcriptomic and proteomic analysis can reveal regulatory mechanisms involving NLR and TLR signaling pathways against NADC30-like PRRSV [13].

Macrophages, as pivotal phagocytes, execute three core interconnected functions—phagocytosis, antigen presentation, and cytokine secretion—that collectively underpin their central role in immune homeostasis. These cells are instrumental in the initiation and propagation of acute inflammatory responses. Upon invasion by exogenous bacterial lipopolysaccharide (LPS), macrophages become activated and release inflammation-associated cytokines; this functional efficacy relies on the induction of oxidative stress, thereby disrupting cellular redox equilibrium. *Cistanche deserticola* polysaccharide (CDP) exhibits unique bidirectional regulatory properties, multidimensionally modulating macrophage immunoreactivity and anti-inflammatory functions. This intervention effectively suppresses LPS-evoked NF-κB nuclear translocation and AP-1 activation, markedly attenuating the secretion of pro-inflammatory mediators, down-regulating inducible nitric oxide synthase (iNOS) expression, and curtailing reactive oxygen species (ROS) overproduction.

In this study, we elucidated the anti-inflammatory activity of CDP-D2-N1 from *Cistanche deserticola* using the transcriptomic profiling of LPS-stimulated RAW264.7 macrophages. Our findings demonstrate that CDP-D2-N1 mitigates LPS-induced inflammatory responses by suppressing pro-inflammatory signaling cascades. These results provide a foundational framework for further investigation into the anti-inflammatory targets and molecular mechanisms of CDP-D2-N1.

## 2. Materials and Methods

### 2.1. Materials and Reagents

CDP-D2-N1 were isolated from *Cistanche deserticola stems* via sequential chromatographic purification with protocols previously established in our laboratory for polysaccharide extraction and isolation [17].

We used lipopolysaccharide (LPS) (Sigma Corporation, St. Louis, MO, USA), RAW264.7 (Chinese Academy of Sciences Cell Bank, Shanghai, China), fetal bovine serum (FBS), culture medium (DMEM), double antibiotic (streptomycin and penicillin) and trypsin (Gibco, Grand Island, NY, USA), TNF-α, IL-6 and IL-1 kits, RNA reverse transcription kits, RNA extraction kits, fluorescence quantification kits (Solebaum Bioengineering Co. Ltd., Beijing, China), RIPA cell lysate, PMSF protease inhibitor, primary antibody dilution (Shanghai Biyuntian Biotechnology Co., Ltd., Shanghai, China), Enzyme microplate reader (Bio-Rad Laboratories, Hercules, CA, USA), Thermal Cycling PCR (Bio-Rad, USA, Hercules, CA, USA), Quantitative PCR (Thermofisher Scientific, Waltham, MA, USA), Nitrogen Purge (Reacti-Therm, Thermo Fisher Scientific, Waltham, MA, USA), Ion Chromatography (ICS 5000+, Thermo Fisher Scientific, Waltham, MA, USA), and a Vortex Mixer (XH–T, Qingdao, China).

### 2.2. Methods

#### 2.2.1. RAW264.7 Cell Culture

RAW264.7 cell culture and treatments were performed according to Jiang et al. [5]. The experimental design and treatment protocol were consistent with those in our previous publications. Cells were seeded in DMEM/F-12 medium (supplemented with 10% fetal bovine serum, 100 U/mL penicillin, and 100 μg/mL streptomycin) and incubated for 24 h. DMEM/F-12 was selected for RAW264.7 culture as it provides additional nutritional support. Subsequently, cells were exposed to lipopolysaccharide (LPS; 10 μg/mL) alone or in combination with varying concentrations of test samples for an additional 12 h. Following treatment, supernatants and cells were harvested for downstream analyses. Cells were allocated into four experimental groups, as follows: normal control (CK) group—untreated cells; model (LPS) group—cells treated with LPS alone; positive control (DSMS) group—cells pretreated with LPS followed by 0.5 μg/mL dexamethasone (DEX) for 12 h; and the sample group—cells pretreated with LPS followed by treatment with CDP-D2-N1 for 12 h. Specifically, LPS-stimulated cells were treated with CDP-D2-N1 at doses of 62.5 μg/mL, 125 μg/mL, and 250 μg/mL, designated as CDP-L, CDP-M, and CDP-H, respectively [17].

#### 2.2.2. CCK-8 Assay

The effect of CDP-D2-N1 on RAW264.7 cell viability was assessed using the Cell Counting Kit-8 (CCK-8) assay [18,19]. The cell treatment protocol was performed according to previously established protocols [17]. Cell viability was determined by measuring the optical density (OD) at 450 nm using a microplate reader. All OD values were blank-corrected (subtracting the mean absorbance of cell-free blank wells), and measurements were performed in triplicate and averaged [20].

$$Cell\;viability\left(\%\right)=\frac{ODsample-OD\;blank}{ODcontrol-OD\;blank}\times 100\%$$

#### 2.2.3. Real-Time Quantitative PCR

The mRNA expression levels of target genes (IL-1β, TLR4, TNF-α, and IL-6) were quantified using quantitative real-time PCR (qPCR). The protocol was adapted from previous studies [21,22] with the following modifications: primer sequences were redesigned (Table 1) and the annealing temperature was optimized to 60 °C. Cells were pretreated with varying concentrations of CDP-D2-N1 for 6 h, followed by LPS (10 μg/mL) stimulation for an additional 12 h. Each experimental group was set up in triplicate. Total RNA was extracted using TRIzol reagent according to our laboratory’s established protocol [21,23,24]. Reverse transcription was performed using a PrimeScript RT kit. Gene expression levels were quantified using the 2^−ΔΔCt^ method, with β-actin serving as the endogenous reference gene for normalization [22,23]. The specific primer sequences used in this study are listed in Table 1.

#### 2.2.4. Total RNA Extraction

Cells from experimental and control groups were washed with PBS, collected in centrifuge tubes containing 1 mL of TRIzol™ reagent, snap-frozen in liquid nitrogen, and stored at −80 °C. Total RNA was extracted using the method established by our laboratory [24]. Briefly, TRIzol™ reagent was used to isolate total RNA, followed by DNase I treatment to eliminate genomic DNA contamination. RNA quality was assessed using an Agilent 2100 Bioanalyzer and a NanoDrop™ 2000 spectrophotometer. Screening criteria required a purity index with A260/280 ratios between 1.8 and 2.2 and A260/230 ratios ≥ 2.0, integrity with an RNA Integrity Number (RIN) ≥ 6.5 and 28S/18S rRNA ratio ≥ 1.0, and a minimum concentration of 1 μg. Only RNA samples meeting these quality standards were used for subsequent sequencing library construction.

#### 2.2.5. cDNA Library Construction and Sequencing

Total RNA was extracted from RAW264.7 cells using TRIzol reagent (Invitrogen, Carlsbad, CA, USA) according to the manufacturer’s protocol. RNA purity and integrity were assessed using a NanoDrop 2000 spectrophotometer (Thermo Fisher Scientific, Waltham, MA, USA) and an Agilent 2100 Bioanalyzer (Agilent Technologies, Santa Clara, CA, USA). Only samples with RNA Integrity Number (RIN) ≥ 7.0 were used for library construction. Poly(A) mRNA was isolated from total RNA using oligo(dT) beads and fragmented into short fragments (≈200 bp) using the fragmentation buffer. First-strand cDNA was synthesized using random hexamer primers and SuperScript II reverse transcriptase (Invitrogen), followed by second-strand cDNA synthesis. The cDNA fragments were end-repaired, A-tailed, and ligated with Illumina sequencing adapters. The ligation products were size-selected (≈300 bp) and PCR-amplified for 10–12 cycles. The amplified libraries were accurately quantified using a TBS 380 fluorescence quantification system (Turner Biosystems, Sunnyvale, CA, USA) and sequenced on an Illumina HiSeq X Ten or NovaSeq 6000 platform (Illumina, San Diego, CA, USA) with paired-end 150 bp (PE150) reads, generating approximately 6–8 GB of raw data per sample. Detailed procedures were performed as described in our previous reports [24].

#### 2.2.6. Position of Genetic Reads

Raw sequencing reads were filtered to remove adapter sequences, low-quality bases (Phred score < 20), and reads containing ambiguous bases (N). The resulting high-quality clean reads were aligned to the mouse reference genome (GRCm38/mm10) using HISAT2 (v2.1.0) with default parameters. The alignment results were sorted and indexed using SAMtools (v1.9). The position of genetic reads on the reference genome was visualized using the Integrative Genomics Viewer (IGV). Detailed read alignment and positioning methods were adapted from Gong et al. [24,25].

#### 2.2.7. Data Handling

To confirm the dependability of the transcriptome sequencing data, raw sequencing data of samples were subjected to strict quality control in this study, which provided a reliable data base for subsequent analyses. Fastp (v0.18.0) was employed to control different quality data. The use of oligo (dT) magnetic beads allowed the enrichment of mRNA containing poly A tails, but small amounts of rRNA may still be present. We used the alignment tool Bowtie 2 (v2.4.4) for sequence segments to match high-quality reads to the rRNA database.

#### 2.2.8. Sample-Related Analysis

To evaluate the consistency of biological replicates within each group, Pearson’s correlation coefficients were calculated based on the expression levels (FPKM values) of all genes across samples. A correlation heatmap was generated using the pheatmap package (v1.0.12) in R (v4.0.3) to visualize pairwise correlations. Additionally, principal component analysis (PCA) was performed using the prcomp function in R to further assess sample clustering and group separation. These analyses ensured the reliability and reproducibility of the transcriptomic data. The methods were adapted from previously described protocols [24,26].

#### 2.2.9. Analysis of Differential Expression

Analysis of the CK, LPS and CDP groups was performed using DESeq2 (v1.42.0) software. Differentially expressed genes (DEGs) were identified with thresholds of |log_2_FC| ≥ 1 and adjusted *p*-value (false discovery rate, FDR) < 0.05. For differentially expressed proteins (DEPs), thresholds of |log_2_FC| ≥ 1 and *p* < 0.05 were applied (without FDR correction, due to the limited number of identified proteins in label-free proteomic datasets). These relatively stringent fold-change thresholds were selected to prioritize biologically meaningful changes and reduce false positives.

#### 2.2.10. Enrichment Analysis

Gene Ontology (GO) enrichment analysis and Kyoto Encyclopedia of Genes and Genomes (KEGG) pathway enrichment analysis were performed using the cluster Profiler package (v3.18.0) in R. Differentially expressed genes (DEGs) with adjusted *p*-value < 0.05 were used as input. For GO analysis, genes were classified into three categories: biological process (BP), cellular component (CC), and molecular function (MF). For KEGG analysis, genes were mapped to reference pathways. Enrichment significance was determined using a hypergeometric test, and *p*-values were adjusted using the Benjamini–Hochberg method (false discovery rate, FDR < 0.05), the Gene Ontology database (GO; http://www.geneontology.org) (accessed on 23 May 2024) and the database of the Kyoto Encyclopedia of Genes and Genomes (KEGG; http://www.genome.jp/kegg/) (accessed on 23 May 2024). Specific enrichment methods can be found in Hou et al. [26].

#### 2.2.11. PCR Validation of Different Gene Expression

Ten genes associated with immunity DEGs were selected for RT-qPCR confirmation. Total RNA was reverse transcribed into cDNA using specific primers and the Prime Script RT kit. Primers for RT-qPCR in different directions are described in Table 1. The reaction system is referred to in existing research [27]. Using the method previously described with slight modifications, the expressions of IL-6, TNF-α, TLR4 and IL-1β were detected. RAW264.7 cells were cultured at a number of 5 × 10^4^ cells in each well for 24 h. Afterwards, the cells were processed with LPS and different concentrations of CDP-D2-N1 for 12 h, with three replicate wells in each group. Finally, expression levels were then assessed by qPCR, with β-actin as a standardized reference. The calculation method employs the 2^−ΔΔCt^ approach [21,23].

### 2.3. Statistical Analyses

All statistical analyses were performed using Origin 2021 (OriginLab Corp., Northampton, MA, USA) and GraphPad Prism 9.0 (GraphPad Software, Boston, MA, USA; accessed on 23 May 2024). Prior to hypothesis testing, the normality of residuals was assessed via the Shapiro–Wilk test, and the homogeneity of variances was assessed via Levene’s test. For comparisons involving three or more groups, one-way ANOVA was applied if parametric assumptions were met; the Brown–Forsythe ANOVA was used for data with unequal variances. Pairwise comparisons between two groups were evaluated using a two-tailed Student’s *t*-test for parametric data or the Mann–Whitney U test for non-parametric data. For all ANOVA models, Tukey’s Honest Significant Difference (HSD) test was used as the post-hoc correction to control the family-wise error rate for multiple comparisons; non-parametric ANOVAs were followed by the Dunn test with Bonferroni adjustment.

All cell-based experimental data are presented as mean ± standard deviation (SD) from *n* = 3 independent biological replicates, defined as cells seeded, treated, and harvested on separate days to account for day-to-day experimental variability. Each biological replicate included 3 technical replicates (parallel wells) per condition; statistical inferences were drawn from biological replicate means, while technical replicates were used solely to estimate intra-assay variability. For reference: CCK-8 viability, phagocytosis, NO production, iNOS mRNA quantification, qPCR, and ELISA cytokine assays each used *n* = 3 biological replicates.

The omics-specific statistical methods are detailed in the corresponding sections; RNA-seq differential expression was analyzed via DESeq2 with Benjamini–Hochberg false discovery rate (FDR) correction (Section 2.2.9).

A *p*-value < 0.05 was considered statistically significant. Significance levels are indicated as * *p* < 0.05, *p* < 0.01, and * *p* < 0.001 in all figures. Every figure legend explicitly defines the statistical test applied and the interpretation of all symbols.

## 3. Results

### 3.1. Effect of LPS Induction on RAW264.7

The effect of LPS on RAW264.7 cell proliferation was evaluated using the CCK-8 assay at concentrations of 1, 10, 20, and 100 μg/mL over 6, 12, and 24 h, with qPCR simultaneously detecting TNF-α and TLR4 mRNA expression to assess inflammatory model feasibility. As shown in Figure 1a,b, cell survival rates in the 1 and 10 μg/mL LPS groups did not differ significantly from the control (*p* > 0.05), whereas the 20 and 100 μg/mL groups showed significant decreases (*p* < 0.05). RAW264.7 cells reached optimal survival rates at 12 h post-LPS induction, indicating this as the ideal time point. Figure 1b displays the cell viability of RAW264.7 macrophages at different time points (6 h, 12 h, 24 h, 36 h, and 48 h) using a box plot. The results indicate that cell viability increased to a peak at 12 h. Subsequently, cell viability gradually decreased over 12 h. Statistical analysis revealed significant differences between 12 h and other time points. These findings suggest that treatment duration significantly affects cell viability, with 12 h showing the highest viability before a gradual decline at later time points. The qPCR results (Figure 1c,d) reveal that LPS significantly up-regulated TNF-α and TLR4 mRNA levels compared with the control, though no clear concentration-dependent trend was observed (coefficient of variation: 15–20%). Notably, TNF-α, as a central inflammatory mediator, is directly triggered by and can be synergistically activated by other inflammatory cytokines [28].

Among these, TNF-α and TLR4 expressions in the 10 μg/mL LPS group were significantly higher than at other concentrations (*p* < 0.001), indicating effective inflammatory induction without excessive cytotoxicity. Notably, the 10 μg/mL LPS concentration was optimized to trigger a robust pro-inflammatory transcriptional response without compromising plasma membrane integrity or causing widespread cell death, establishing a physiologically relevant inflammatory activation model suitable for evaluating immunomodulatory rather than cytoprotective effects. Based on cell survival rates and gene expression data, 10 μg/mL LPS for 12 h was selected as the optimal condition for establishing the RAW264.7 immune damage model [19,29]. Subsequent experiments used CDP-D2-N1 concentrations of 62.5, 125, and 250 μg/mL based on prior laboratory research [17].

### 3.2. Effects of Different CDP-D2-N1 Treatment on RAW264.7 Proliferation

After 24 h of cell attachment, cells were treated with CDP-D2-N1 at different concentrations for 6, 12, 24, 36, or 48 h. At the tested concentrations, pretreatment affected cell viability at different culturing durations. As shown in Figure 2, with increasing culture time, all polysaccharide samples exhibited higher cell viability at 12 h compared to other time points. Concurrently, RAW264.7 cells exhibited peak viability at 12 h of culture. This peak was reached because prolonged culture duration diminished cell viability. The CCK-8 assay assessed the effects of CDP-D2-N1 (15.625–1000 μg/mL) on RAW264.7 cell growth. As depicted in Figure 3, CDP-D2-N1 concentrations of 62.5, 125, and 250 μg/mL resulted in a RAW264.7 cell viability of 108.7 ± 4.0%, 123.0 ± 5.6%, and 134.6 ± 6.9%, respectively, which values are significantly higher than those of other concentrations (*p* < 0.05). Therefore, in subsequent experiments, concentrations of 62.5, 125, and 250 μg/mL were selected as the low-, medium-, and high-treatment doses of CDP-D2-N1.

### 3.3. CDP-D2-N1 Significantly Suppressed the Expression of TNF-α, IL-1, and IL-6

As shown in Figure 3a–c, compared with the LPS-induced inflammatory cell model group, the expression levels of TNF-α, IL-1β, and IL-6 were markedly reduced in the 31.25 μg/mL CDP-D2-N1 pretreatment group. CDP-D2-N1 markedly inhibited the expression of three pro-inflammatory cytokines, namely, TNF-α, IL-1, and IL-6. However, these inflammatory markers were still higher than those in the negative control (NC) group. Macrophages can initiate innate immune responses to fight infection and inflammation, and are key cells in immune system research. The modulatory effect of natural polysaccharides on immune activity can also be achieved by regulating macrophage immune function [30,31]. Changes in cell viability were monitored by the CCK-8 assay to evaluate the effect of CDP-D2-N1 on RAW264.7 proliferation. As shown in Figure 3d, CDP-D2-N1 exhibited a promotional effect on cell growth. Regarding the NC group, the cell viability appeared slightly lower over the 48 h period. This observation is primarily attributed to normal experimental variability and the specific initial seeding density/culture conditions, rather than reduced cell viability or cytotoxicity. Given the relatively short doubling time of RAW264.7 cells, the cells may have reached a high confluence state by 48 h, which can plateau or slightly alter the metabolic readout of the CCK-8 assay. No obvious cytotoxicity was observed in any group. The effects of polysaccharides on cell viability are mainly attributed to their structural characteristics; the monosaccharide composition, molar ratios, and molecular weights of polysaccharides all influence cell growth and vitality.

### 3.4. Effects of CDP-D2-N1 Pretreatment on LPS-Induced Interleukin Gene Expression in RAW264.7 Macrophages

Pro-inflammatory cytokines such as TNF-α, IL-6, and IL-1 are produced by macrophages and mediate inflammatory responses. As shown in Figure 4a, after 12 h of pretreatment in RAW264.7 cells, CDP-L, CDP-M, or CDP-H was added, followed by stimulation with 10 μg/mL LPS. The results show no statistically significant difference in cell viability compared with the normal control group (CK) (*p* > 0.05), confirming that the LPS-induced protocol and CDP pretreatment did not cause significant toxicity. The results indicate that, compared with the CK group, the mRNA expression levels of IL-1, TNF-α, and IL-6 were significantly elevated in the 10 μg/mL LPS-treated group (*p* < 0.05), confirming the successful induction of an inflammatory response (Figure 4b–d). Following pretreatment with different doses of CDP-D2-N1, the expression levels of IL-1, TLR4, TNF-α, and IL-6 were significantly lower than those in the LPS-only group (*p* < 0.05). This finding is consistent with Liu’s study, which showed that the polysaccharide SIP-II alleviates LPS-induced inflammatory responses by inhibiting the secretion of IL-6 and TNF-α [20].

### 3.5. Effect of LPS and CDP-D2-N1 Treatment on RAW264.7 Toll-like Receptor TLR4

Polysaccharides, as exogenous additives, need to pass through cell surface receptors in order to function in cells. Toll-like receptors on the surface of immune cells are usually polysaccharide-binding receptors, among which TLR4 is an important polysaccharide receptor that regulates the immune response of immune cells through the NF-κB signaling pathway, thus stimulating the production of cytotoxic factors by immune cells [32]. As shown in Figure 5a, the expression of intracellular TLR4 was markedly increased (*p* < 0.05) in LPS and 31.25 μg/mL CDP-D2-N1-treated RAW264.7 compared to the CK group. The expression of TLR4 in RAW264.7 after CDP-D2-N1 treatment was significantly increased (*p* < 0.05) compared to the LPS-treated group, but there was no salient difference between the CDP-D1- and CDP-D2-N1-treated groups (*p* > 0.05).

### 3.6. Effect of CDP-D2-N1 Pretreatment on LPS-Induced Phagocytosis of RAW264.7

LPS and CDP-D2-N1 act as exogenous additives to RAW264.7 and RAW264.7 removes exogenous invaders by phagocytosis, thus exerting its role in regulating immunity. CDP-D2-N1 had a certain effect on the phagocytosis ability of RAW264.7 induced by LPS. As shown in Figure 5b, RAW264.7 phagocytosis in both LPS-induced and LPS + CDP-treated groups was higher than that in the CK group, and RAW264.7 phagocytosis in the LPS + CDP-treated group reached a highly significant level (*p* < 0.001). Compared with the LPS-induced group, RAW264.7 phagocytosis was significantly higher in the CDP-D2-N1-pretreated RAW264.7, and RAW264.7 phagocytosis in the CDP-H-pretreated RAW264.7 reached a highly significant level (*p* < 0.01).

### 3.7. Effect of CDP-D2-N1 Pretreatment on LPS-Induced NO Production in RAW264.7

One of the important defense mechanisms of macrophages is the production of NO, so evaluating the effect of CDP-D2-N1 on the NO production of RAW264.7 can indirectly regulate macrophage immunoreactivity by CDP-D2-N1. From Figure 5c, we see that in contrast with the CK group, the production of NO in macrophages after CDP-D2-N1 pretreatment was increased, and the LPS-induced, CDP-L-pretreated, and CDP-M-pretreated groups reached a highly distinct level (*p* < 0.01); the variation between the two is not dramatic (*p* > 0.05), although the production of NO in RAW264.7 was increased after CDP-H treatment. In contrast to the LPS-induced group, the NO production of RAW264.7 was significantly reduced after CDP-D2-N1 pretreatment, with the reduction reaching a highly significant level in the CDP-L treatment group (*p* < 0.01), and very significant reductions were seen in the CDP-M and CDP-H treatment groups (*p* < 0.001). This shows that CDP-D2-N1 modulates macrophage immunoreactivity by reducing NO production in RAW264.7.

### 3.8. Effect of CDP-D2-N1 Pretreatment on LPS-Induced iNOS Gene Expression in RAW264.7

iNOS can be induced to be expressed in cells to regulate the anti-inflammatory and antibacterial activities of RAW264.7 by controlling NO synthesis [33]. As can be seen from Figure 5d, the iNOS gene expression of RAW264.7 was elevated to a highly significant level after LPS induction (*p* < 0.001), compared with which, after CDP pretreatment, the iNOS gene expression showed a decreasing trend and a dose-dependent pattern, in which the decrease in iNOS gene expression of RAW264.7 in the CDP-H pretreatment group was highly significant (*p* < 0.001), and the decrease after CDP-M pretreatment was at a highly significant level (*p* < 0.01), but there was no apparent variation in the CDP-L pretreatment group, and the decrease was not significant (*p* > 0.05). The results show that CDP-D2-N1 could achieve the regulation of NO molecules by modulating the expression of iNOS genes in RAW264.7, thus regulating the efficacy of RAW264.7.

### 3.9. Validation of LPS-Induced RAW264.7 Transcriptome Sequencing Data and Quality Control

To investigate the differential gene expression in RAW264.7 cells pretreated with CPD versus those induced by LPS, we collected nine RAW264.7 samples, including normal control (CK), LPS-induced (LPS), and CDP-H pretreatment + LPS-induced (CDP). After sequencing RAW264.7, the raw data of each sample are shown in Table 2. The data distribution was 41–87 million bases. After filtering, the rate of Q20 bases was higher than 97%, the rate of Q30 bases was in the range of 94.14–95.37%, and the GC content was above 45%. The pre-processing results of the raw data were good, providing a reliable data basis for the subsequent bioinformatics analyses, such as differential expression analysis, functional annotation and pathway enrichment [34].

### 3.10. Analysis of Transcriptome Sequencing Data and Regional Distribution Statistics

In this experiment, nine libraries were constructed with three replicates each of CK, LPS-induced and CDP-pretreated groups. The library was sequenced on the Illumina Hi Seq™ 4000. A total of 517,003,036 raw bases were obtained. After quality control, high-quality (HQ) bases were obtained at 99.95% of the original reads. In this study, based on the reference sequences of existing cells, the QC-treated pure reads were compared with the reference genes and the reference genome, and the comparison results are presented in Table 3. The RNA sequence coverage of all samples reached more than 95%, which indicates that the sequencing data had a high degree of targeting and integrity, and no significant contamination signals were detected. Among them, total reads represent the amount of raw sequencing data, clean reads indicate the amount of valid data after quality filtering, mapped reads are the number of samples successfully aligned to the reference genome, and unique mapped reads refer to the quantity of samples with a unique alignment location on the reference genome. High-quality alignment results provide a reliable basis for subsequent transcript assembly and quantitative analysis.

### 3.11. Differential Gene Expression Analysis

Pearson correlation coefficients were calculated to help in assessing sample reproducibility, as displayed in Figure 6. The correlation coefficients between parallel samples were all above 0.77, and Fisher’s Z-transformation test confirmed that intra-group correlations were significantly higher than inter-group correlations (*p* < 0.05). The findings indicate acceptable repeatability among parallel samples. It should be noted that the correlation coefficients (>0.77) are somewhat lower than the >0.9 typically observed in clonal cell lines; this likely reflects the inherently higher biological variability of primary macrophage cultures. No outliers were observed. The cluster analysis of differentially expressed genes can effectively reflect the dynamic change characteristics of RNA sequences among different experimental groups. The results are shown in Figure 6. Up-regulated genes are signaled in red, down-regulated genes in blue. In contrast to the CK, the total number of variably expressed genes (16 up-regulated and 3 down-regulated) in the LPS-induced group was 19, whilst the CDP pretreatment group yielded a total of 226 variations (46 up-regulated and 180 down-regulated) (Figure 6A). There were 210 genes being differentially expressed in the CDP-pretreated group and the LPS-induced group, respectively, of which 17 were up-regulated genes and 193 were down-regulated genes (Figure 6B). The numbers of DEGs in each group of CDP vs. LPS, CDP vs. CK and LPS vs. CK (Figure 6C) were calculated and graphed into a Venn diagram, with CDP, CK and LPS RAW samples sharing one DEG (Figure 6D,E).

It is noted that the number of DEGs in the LPS vs. CK comparison is relatively modest, which likely reflects the stringent |log_2_FC| ≥ 1 threshold applied and the 12 h time point, which captures a later phase of the transcriptional response following the initial robust activation. This interpretation is supported by the strong functional responses (cytokine induction, NO production, phagocytosis) observed in the same cells.

### 3.12. Differential Gene KEGG Enrichment Analysis

KEGG function enrichment analysis was performed by analyzing 455 DEGs. The results are shown in Figure 7, according to which 425, 406, and 122 signaling pathways were activated or inhibited in CDP vs. LPS, CDP vs. CK, and LPS vs. CK, respectively, including 40, 22, and 5 cellular process pathways, 73, 69, and 19 environmental information entry pathways, 2, 9, and 0 genetic information pathways, 130, 137, and 65 human disease pathways, 54, 66, and 2 metabolic pathways, and 126, 103, and 31 biosystems pathways. KEGG down-regulation was enriched in the TNF signaling pathway, cytokine–cytokine receptor interaction, and the chemokine signaling pathway in the CDP group compared to the CK group; KEGG up-regulation was enriched in fatty acid metabolism and steroid biosynthesis. KEGG down-regulation was enriched in the AMPK signaling pathway in the CDP group in comparison to the LPS group, KEGG down-regulation was enriched in the AMPK signaling pathway, as well as in antigen processing and presentation, steroid biosynthesis, phagolysosomes, the Hippo signaling pathway, and fatty acid metabolism. KEGG up-regulation in the LPS group versus the CK group was enriched in cytokine–cytokine receptor interactions, the chemokine signaling pathway, NOD-like receptor, NF-kappa B, TNF and the Toll-like receptor signaling pathway. RAW264.7’s main functions include phagocytosis, antigen presentation, the secretion of inflammatory factors, and metabolic reprogramming. Therefore, the KEGG pathway needs to be linked to these functions. The up-regulation of antigen processing and presentation pathways may enhance the antigen presentation capacity of macrophages and promote T cell immune response. The up-regulation of phagosome-associated pathways may enhance phagocytic efficiency and contribute to the fight against infection. The AMPK and the PPAR signaling pathways are commonly associated with metabolic regulation, and may affect macrophage energy metabolism and polarization status, thereby modulating the inflammatory response. The phagosome and antigen processing and presentation pathways are clearly directly related to the immune function of macrophages, while pathways like the AMPK signaling pathway and fatty acid metabolism are involved in metabolic regulation, and may affect macrophage energy status and polarization. Based on pathway function, differential entries can be categorized into immune core pathways, metabolic and biosynthetic pathways, disease-specific pathways, neurological and developmental-related pathways, and other key pathways. Among these, there are five immune core pathways, mainly including the following: antigen processing and presentation; RAW264.7 activates adaptive immune response by presenting pathogen antigens to T cells through MHC-I/MHC-II molecules; CDP enhances the antigen presentation capacity of macrophages, promotes Th1/phagosome formation, lysosome fusion, and pathogen degradation (acid phosphatase activity), and enhances the macrophage clearance of bacteria, viruses, or parasites. The AMPK signaling pathway (AMPK), which regulates metabolic reprogramming and inflammatory responses via the energy sensor AMPK, promotes macrophage polarization from pro-inflammatory (M1) to reparative (M2) so as to alleviate chronic inflammation, and reduces lipid accumulation via inhibition of the mTOR pathway PPAR signaling pathway (PPARα/γ/δ). PPARα/γ/δ regulates fatty acid metabolism and the expression of inflammatory factors and cell differentiation through nuclear receptors, and the activation of PPARγ can inhibit NF-κB activity, reduce IL-6 and TNF-α secretion, and promote M2 polarization. Inflammatory Bowel Disease (IBD) involves an imbalance in the intestinal immune microenvironment, and can reduce intestinal inflammation by inhibiting M1 polarization or promoting M2 polarization.

### 3.13. Differential Gene GO Functional Annotation Analysis

The GO enrichment analysis of LPS induction combined with CDP pretreatment to alter the function of DEGs and their associated biological processes in RAW264.7 revealed the remarkable enrichment of DEGs in the entries of cellular processes, catalytic activities, and cellular parts (Figure 7D–F). The potential functions of 455 DEGs were investigated by GO analysis. The biological processes (BP) category mainly includes DEGs associated with cellular processes, individual tissue processes, and metabolic processes; the molecular functions (MF) category mainly consists of DEGs associated with bound and catalytic activities; and the cellular components (CC) category mainly consists of DEGs associated with cellular and cellular fraction-related differential genes. The CK, CDP and LPS groups had larger values of chemokine ligand 10 (CXCL10), transmembrane protein four domain A7 (MS4A7), and interferon regulatory factor 7 (IRF7), |log2 fold change| > 2. Meanwhile, the CDP and CK groups were gathered together and are very different from the LPS group. The BP process of CDP vs. LPS was analyzed using GO enrichment, and six significant GO entries (cell aggregation, rhythmic process, cell killing, cell proliferation, growth, behavior) were obtained.

Some entries relate to cell aggregation, rhythmic processes, cell killing, proliferation, growth and behavior. For RAW264.7, these are processes closely related to immune function. The significance of each GO entry is analyzed in relation to the function of RAW264.7. Cell aggregation may be involved in phagocytosis and inflammatory foci formation, while rhythmic processes may be related to metabolic rhythms or the cell cycle. CDP-D2-N1, as an active ingredient, is often immunomodulatory, and these pathways influence the function of RAW264.7. Cell aggregation promotes macrophage–macrophage junctions by up-regulating cell adhesion molecules or extracellular matrix proteins, and the aggregated RAW264.7 can synergistically remove pathogens, thereby enhancing phagocytosis, and also aggregate RAW264.7, which may induce localized inflammatory microenvironments and amplify immune responses. Rhythmic process may involve cell cycle rhythms or metabolic rhythms, and abnormal macrophage rhythms may lead to hyperinflammation or inflammatory activation; CDP may improve RAW264.7 by modulating clock genes or mitochondrial biosynthesis. CDP can improve the metabolic rhythm of RAW264.7 by regulating Clock genes or mitochondrial biosynthesis to alleviate chronic inflammation; cell killing is dependent on reactive oxygen species, lysosomal enzymes, or apoptosis-inducing factors, and CDP can elevate ROS production through the activation of the Nrf2/ARE pathway or by inducing apoptosis-associated gene expression through the TLR4/NF-κB pathway. Cell proliferation can be promoted by the up-regulation of cell cycle proteins or growth factor receptors. However, this study did not conduct the targeted functional verification of these pathways; their regulatory relationship with CDP-D2-N1 is only a predictive clue derived from omics enrichment, and requires further experimental confirmation.

The immune function of RAW264.7 was also correlated with cellular components. In the CDP vs. LPS group, based on the type of differential entry for cellular components, the differences are mainly centered on extracellular structures and junctions, as well as cell membranes and internal components. Among them are virus-associated structures, reflecting the CDP regulation of virus recognition or clearance, the RAW264.7 degradation of viral particles by phagocytosis or lysosomal degradation, and intercellular junction and signaling hubs (cell junction/synapse), which involve tight junctions, cell junctions, and cell junction/synapse, gap junctions or immune synapses. CDP can enhance synergistic resistance to infection by enhancing intercellular communication; the extracellular region/extracellular region includes the extracellular matrix and proteins, and the cell junction/synapse involves the cell junctions and signaling hubs. The extracellular region includes extracellular matrix proteins, chemokines, and pathogen-recognition receptors, which recognize pathogen-associated molecular patterns, such as LPS, and trigger the NF-κB pathway, while CDP enhances innate immune sensitivity through the enhancement of PAMPs recognition. The membrane structure and internal organelle include lysosomes. At the same time, they also relate to organelle functions such as lysosomes, mitochondria, and the endoplasmic reticulum, which can be up-regulated through the up-regulation of lysosomal membrane-enclosed lumen-associated genes to enhance pathogen degradation and mitochondrial ATP production, so as to support the energy requirements of RAW264.7 proliferation and phagocytosis. Protein-containing complexes and molecular machines involve signaling complexes, phagosome-forming complexes, or lipid raft structures, where MAPK complex activation promotes the expression of inflammatory factors, and Rab GTPases orchestrate phagosome–lysosome fusion, affecting pathogen clearance efficiency.

Differentially expressed genes (DEGs) were identified based on two-by-two comparisons between the three groups (CK vs. LPS, CK vs. CDP, LPS vs. CDP). Gene ontology enrichment analysis screened the three significant pathways with the highest numbers of differential genes as signal transduction, immune system and infectious diseases. In combination with the results related to pro-inflammatory factors, Toll-like receptors, signaling molecules NO, and iNOS in RAW264.7 cells, the relevant pathways involved in KEGG enrichment analysis, cytokine–cytokine receptor interactions, the NF-kappa B signaling pathway, and the Toll-like receptor signaling pathway were considered together. It is hypothesized that CDP-D2-N1 may exert immunoregulatory effects via the MAPK signaling pathway.

CDP is involved in the immune and inflammatory regulation of RAW264.7; cytokine and chemokine signaling pathways mediate the expression of pro-inflammatory factors; the TNF signaling pathway (Figure 8) directly inhibits TNF-α-induced NF-κB activation and reduces inflammatory factor release; the TGF -β signaling pathway balances pro-inflammatory and anti-inflammatory responses and promotes tissue repair; the AMPK signaling pathway inhibits mTOR and NF-κB through metabolic reprogramming, and exerts dual anti-inflammatory and metabolic regulatory effects. Combining the results of GO functional exegesis and KEGG enrichment analysis, we see that the TNF signaling pathway was the RAW 264.7 anti-inflammatory and immunomodulatory pathway induced by CDP on LPS.

### 3.14. Validation of Transcriptomics Results

As shown in Figure 9A, the qRT-PCR results of 10 randomly picked differential genes, MT2A, CIART, HJV, CIB2, DNAH1, TBCEL, LPIN1, ABCA1, TMPRSS9, and SPATC1L, are highly concordant with the RNA-seq results, with the same regulatory trend, which confirms the credible applicability of the transcriptome data.

### 3.15. Effect of SB203580 on the TNF Signaling Pathway

The effects of p38 inhibitor (SB203580) pretreatment on LPS-induced RAW264.7 inflammatory factors are shown in Figure 9B. SB203580 is a well-established pharmacological inhibitor of p38 MAPK activity; it does not directly inhibit upstream kinases such as MAP3K6 or MAP2K3/MAP2K6. The difference in the levels of IL-6 and TNF-α produced by RAW264.7 after SB203580 pretreatment followed by LPS induction was highly significant (* *p* < 0.001) compared with the LPS-induced group, but not significant (* *p* > 0.05) in comparison with the CK group. The findings indicate that pretreatment with the p38 inhibitor (SB203580) inhibited RAW264.7 inflammatory factor expression, providing supportive evidence for the involvement of the p38 MAPK branch in LPS-induced inflammation. However, this experiment does not validate the upstream MAP3K6 node, which was identified through transcriptomic KEGG enrichment as a candidate upstream regulator, and requires direct validation in future studies. The canonical TNF-MAPK signaling cascade proceeds as follows: the TNF receptor binds to its ligand, recruiting TRADD, RIP1, and the IKK complex, which activates both the NF-κB pathway and the MAPK pathway. At the MAP3K level, MAP3K6 (also known as ASK2) phosphorylates and activates the downstream MAP2Ks, including MAP2K3 and MAP2K6 (MKK3 and MKK6). These MAP2Ks then phosphorylate and activate p38 MAPK, which in turn phosphorylates downstream transcription factors such as AP-1 and ATF2, promoting the expression of pro-inflammatory cytokines including IL-6, IL-8, and TNF-α. Transcriptomic analyses identified MAP3K6 as a candidate hub molecule in this cascade that was down-regulated by CDP-D2-N1 pretreatment; however, this finding is derived from KEGG enrichment, and has not been directly validated at the protein level in the present study.

After CDP-D2-N1 treatment, the inflammatory response of RAW264.7 in response to LPS stimulation was suppressed, as evidenced by a decrease in IL1, IL6, and TNF-α, an increase in phagocytosis, a down-regulation of TRL4 receptor expression, and a decrease in NO production. Transcriptome analysis indicates that the differential genes were primarily enriched in pathways related to anti-inflammatory and metabolic regulation, such as PPARγ and AMPK pathways, whereas KEGG analysis may show the altered activation status of the TNF signaling pathway and related pathways. Together, these changes support the conclusion that *Cistanche deserticola* polysaccharides exert anti-inflammatory and immunomodulatory effects by regulating macrophage function through multiple pathways. CDP-treated RAW264.7 showed down-regulated expressions of IL-1β, IL-6, and TNF-α and markedly increased LPS-induced pro-inflammatory factor secretion, and CDP may have inhibited AP by reducing the expression of the TLR4 receptor or by blocking the nuclear translocation of NF-κB.1 and NF-κB-driven inflammatory gene transcription, as well as indirectly inhibiting NF-κB activity through the activation of the PPARγ or AMPK pathway. CDP inhibits the downstream inflammatory cascade response by decreasing TLR4 expression and attenuating LPS recognition and signaling. CDP reduces iNOS transcription and decreases NO production by regulating the NF-κB pathway.

Liu et al., in their study of the protective effects of CDP on OGD/RP-induced PC12 cell injury, found that up-regulating the expression of the DJ-1 protein (Parkinson’s disease-associated protein) effectively attenuates the OGD/RP-induced PC12 cell injury. CDP improves the cell survival rate, reduces cell membrane damage, maintains intracellular calcium homeostasis, and prevents the loss of mitochondrial membrane potential (MMP). CDP also reduces apoptosis, inhibits oxidative stress, and promotes the translocation of DJ-1 protein to mitochondria. The DJ-1 protein has been proposed to be involved in neuroprotection and the modulation of mitochondrial integrity in recent years [1]. Cho et al. found that *Korean Erythrina kurkulensis* root extract effectively inhibited LPS-induced inflammation. EKRE significantly reduced the inflammation induced by LPS. EKRE significantly decreased LPS-stimulated IL-6 production, and inhibited the activation of NF-κB and MAPK signaling pathways in RAW264.7. The oral administration of EKRE (50 or 200 mg/kg) significantly attenuated carrageenan-induced edema in the paws of mice, and the high-dose group showed significantly reduced levels of IL-6 and TNF at the site of inflammation. Although the overall changes in IL-6 and TNF in the tissues were not significant, the results suggest that EKRE regulates the inflammatory pathway through multiple targets [35]. Peng et al. found that AP-1 was non-toxic to RAW264.7, and in the LPS-induced inflammation model, AP-1 could effectively inhibit NO release, regulate ROS levels, and significantly down-regulate the mRNA expression of TNF-α, IL-1β and IL-6 [36]. Shi et al. found that p38 mitogen-activated protein kinase (MAPK) played a critical role in the LPS-induced inflammatory signaling pathway in RAW264.7, and that LPS stimulation markedly enhanced TNF-α and IL-6 production [37]. The addition of SB203580 significantly blocked LPS-induced TNF-α production in RAW264.7 and abdominal RAW264.7, but the inhibitory effect on IL-6 was cell-type-specific, significantly inhibiting IL-6 production in RAW264.7, while no significant inhibition was observed in abdominal RAW264.7, or even at high concentrations of SB203580. Instead, IL-6 production was increased, and although LPS dramatically up-regulated the mRNA transcript levels of TNF-α and IL-6 in both cells, SB203580 pretreatment significantly down-regulated the mRNA levels of both.

In summary, polysaccharides show promising medicinal uses by modulating inflammation and cellular responses through multiple pathways. Future research on CDP needs to be combined with modern biotechnology and traditional medicine theory to promote its translational application in the clinic. Notably, the present study only verified the functional role of p38 via the chemical inhibitor SB203580, and we did not perform Western blot detection to quantify the protein expressions of MAP3K6, phosphorylated p38, NF-κB p65 or HIF-1α. The regulatory relationship between CDP-D2-N1 and MAP3K6 at the translational level needs to be further confirmed by protein immunoblotting, MAP3K6 gene silencing, or knockout loss-of-function experiments in follow-up research.

## 4. Discussions

This study investigated the immunomodulatory effects of CDP-D2-N1, a polysaccharide from *Cistanche deserticola*, on LPS-stimulated RAW264.7 macrophages using cellular, transcriptomic, and proteomic approaches. CDP-D2-N1 pretreatment attenuated LPS-induced inflammation by reducing pro-inflammatory cytokines (TNF-α, IL-1β, IL-6), NO production, and iNOS expression, while enhancing phagocytosis and cell viability. These effects were linked to the modulation of the TNF signaling pathway and metabolic reprogramming toward aerobic glycolysis.

The anti-inflammatory activity aligns with prior reports on polysaccharides from *Cistanche deserticola* and other medicinal plants. Transcriptomic KEGG analysis identified the TNF-MAPK cascade as a key pathway, with the down-regulation of MAP3K6 (ASK2) observed in CDP-D2-N1-treated cells. The pharmacological inhibition of p38 using SB203580 reduced IL-6 and TNF-α expression, supporting p38 involvement. However, the proposed TNF–MAP3K6-p38 axis remains hypothetical, as MAP3K6 identification derives solely from transcriptomic enrichment, and SB203580 targets p38 directly—not MAP3K6. Validation via Western blot, siRNA, or knockout experiments is required.

It should also be noted that transcriptomic and proteomic analyses were performed only on the highest dose (CDP-H, 250 μg/mL), while functional assays (CCK-8, qPCR, NO, phagocytosis) were evaluated across all three doses (62.5, 125, and 250 μg/mL). Therefore, the mechanistic insights derived from omics data are specifically applicable to the CDP-H dose; the anti-inflammatory effects observed at lower doses (CDP-L and CDP-M) were confirmed at the functional level but lack corresponding mechanistic data at the omics level.

## 5. Conclusions

CCK-8 and qPCR assays were employed to establish the effects of LPS on RAW264.7 cells (10 μg/mL LPS for 12 h), based on cell viability, TLR4, and TNF-α expression. By evaluating the effects of CDP-D1 and CDP-D2-N1 on cell viability, pro-inflammatory cytokines (TNF-α, IL-1β, IL-6), and Toll-like receptor TLR4, CDP-D2-N1 was identified as a candidate with significant anti-inflammatory effects against LPS-induced injury, and was selected for subsequent mechanistic studies.

The optimal concentrations (62.5, 125, and 250 μg/mL) and treatment duration (12 h) of CDP-D2-N1 for rescuing immune-impaired RAW264.7 cells were determined using CCK-8 and qPCR. Pretreatment with CDP-D2-N1 enhanced the phagocytic capacity of LPS-injured macrophages, suppressed the expressions of TNF-α, TLR4, IL-6, and IL-1β, and consequently improved cellular immune function against LPS-induced injury, with the highest dose (CDP-H) exhibiting the greatest efficacy.

Analysis using transcriptomics technology revealed that 17 differentially expressed genes were up-regulated and 193 differentially expressed genes were down-regulated after the pretreatment of RAW264.7 with CDP-D2-N1, which was inferred to indicate the regulation of the LPS-induced immune injury of RAW264.7 by CDP-D2-N1 through the TNF signaling pathway, via a combination of GO functional annotation and KEGG enrichment analysis. The suppression of IL-6 and TNF-α expression by p38 (SB203580), an inhibitor of its key regulatory enzyme MAP2K3/MAP2K6, also confirms the regulation of the activity of the TNF signaling pathway on immune injury in RAW264.7.

## Figures and Tables

**Figure 1:**
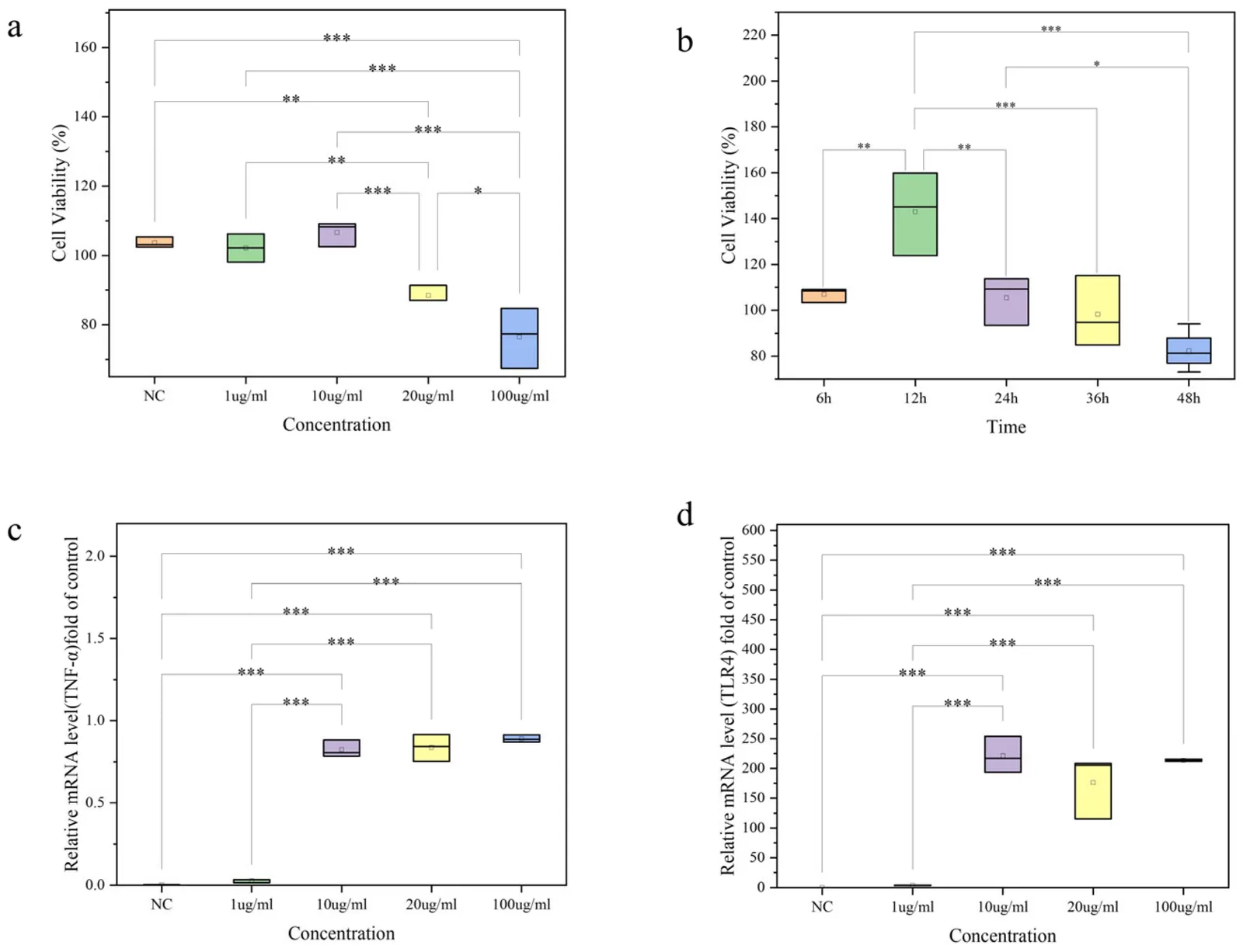
Effects of LPS on RAW264.7 cell viability in terms of concentration (**a**) and time (**b**), as well as the relative mRNA levels of TNF-α (**c**) and TLR4 (**d**) under LPS applied at different concentrations (* *p* < 0.05 ** *p* < 0.01 *** *p* < 0.001).

**Figure 2:**
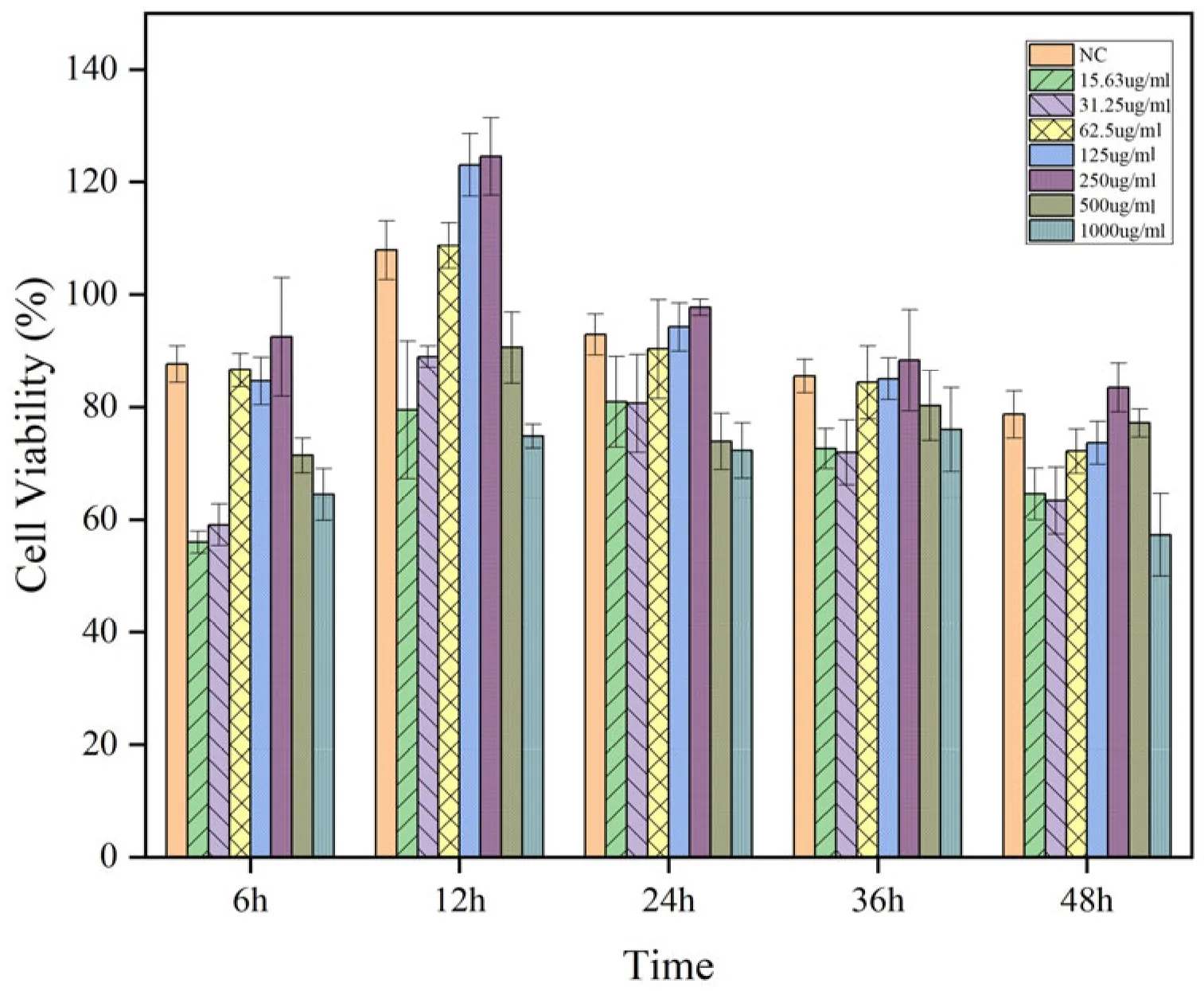
Effects of RAW264.7 on cell viability under CDP-D2-N1 at different concentrations and different treated times.

**Figure 3:**
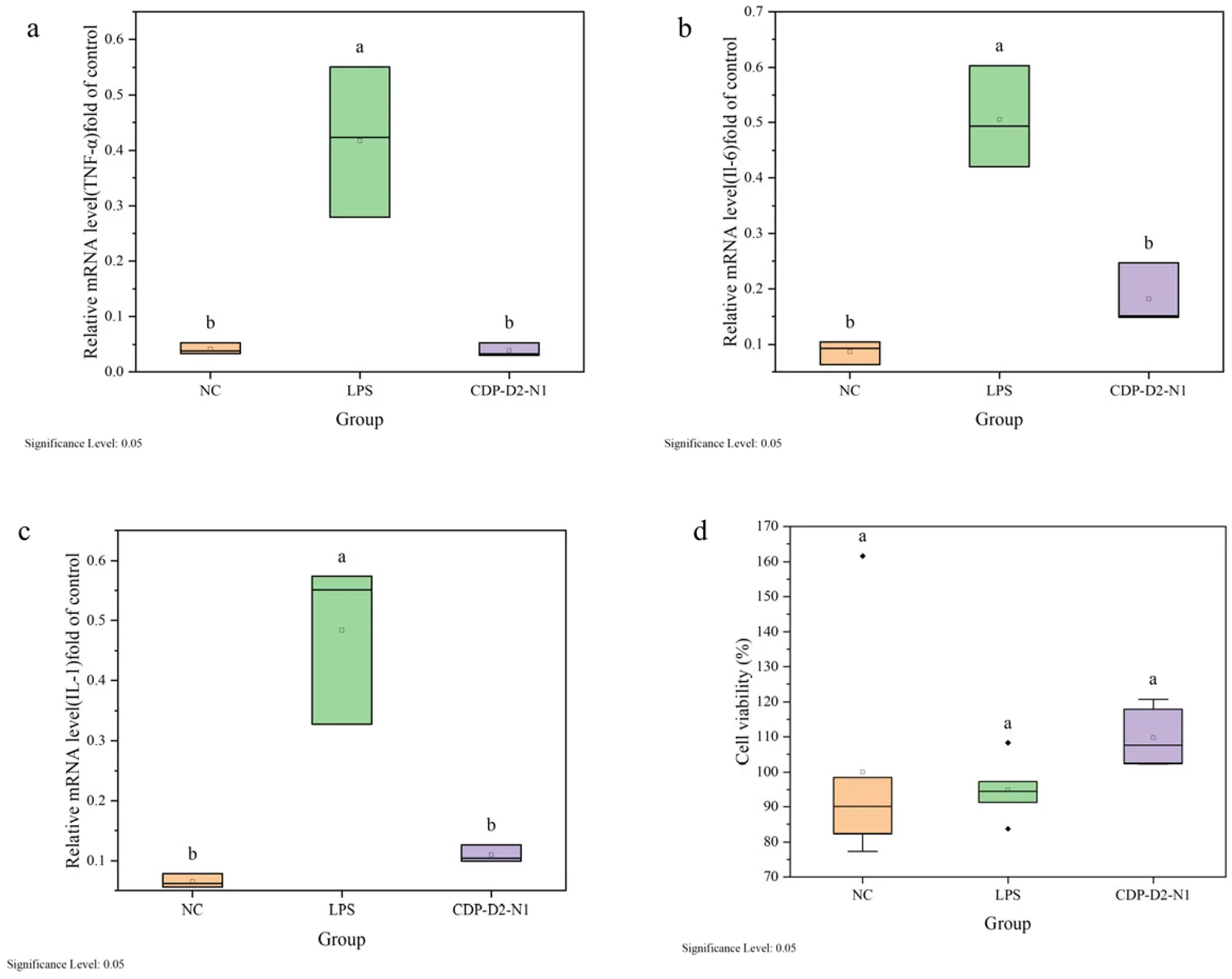
Effects of RAW264.7 on the relative mRNA levels of TNF-α (**a**), IL-6 (**b**), IL-1 (**c**) and cell viability (**d**) under LPS and CDP-D2-N1 at the same doses of combined LPS. Different lowercase letters (**a**,**b**) above bars indicate significant differences between groups (*p* < 0.05).

**Figure 4:**
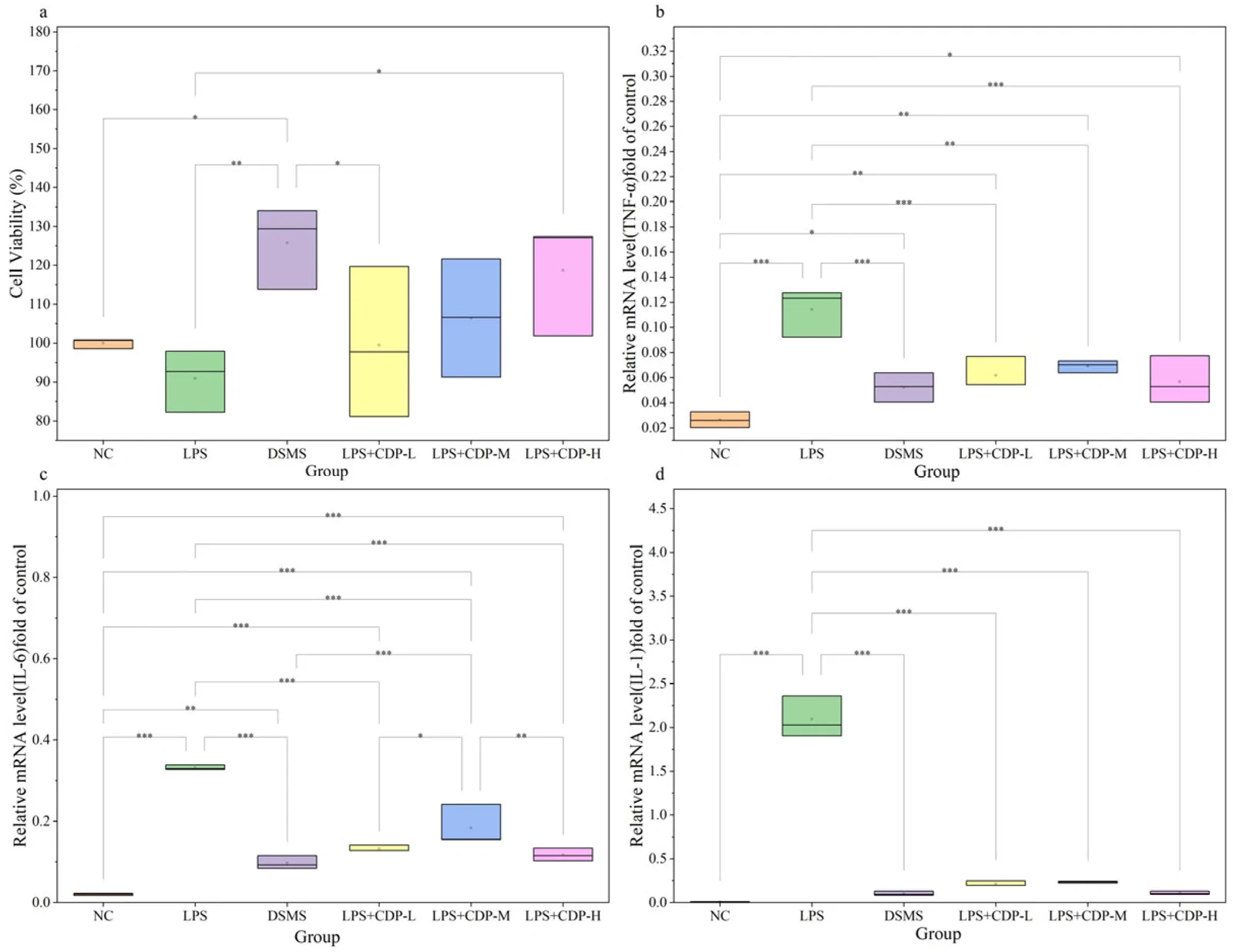
Effects of RAW264.7 on cell viability (**a**), relative mRNA level of TNF-α (**b**), IL-6 (**c**) and IL-1 (**d**) under pretreatment with CDP-D2-N1 at different doses of combined LPS (* *p* < 0.05 ** *p* < 0.01 *** *p* < 0.001).

**Figure 5:**
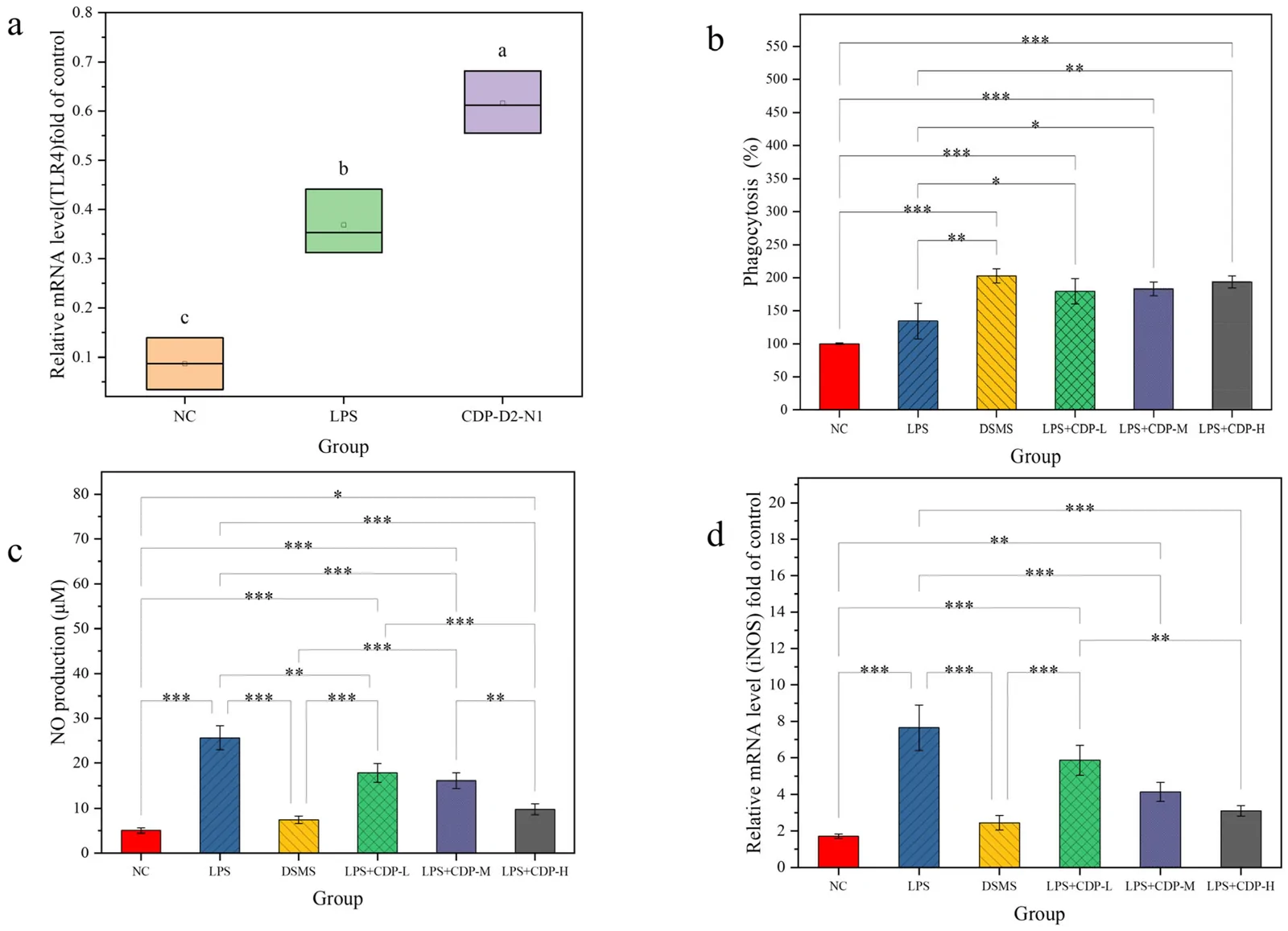
Effects of RAW264.7 on the relative mRNA level of TLR4 (**a**), cell phagocytosis (**b**), NO (**c**), and iNOS (**d**) under pretreatment with CDP-D2-N1 at different doses of combined LPS. Different lowercase letters (a, b and c) above bars indicate significant differences among groups (* *p* < 0.05 ** *p* < 0.01 *** *p* < 0.001).

**Figure 6:**
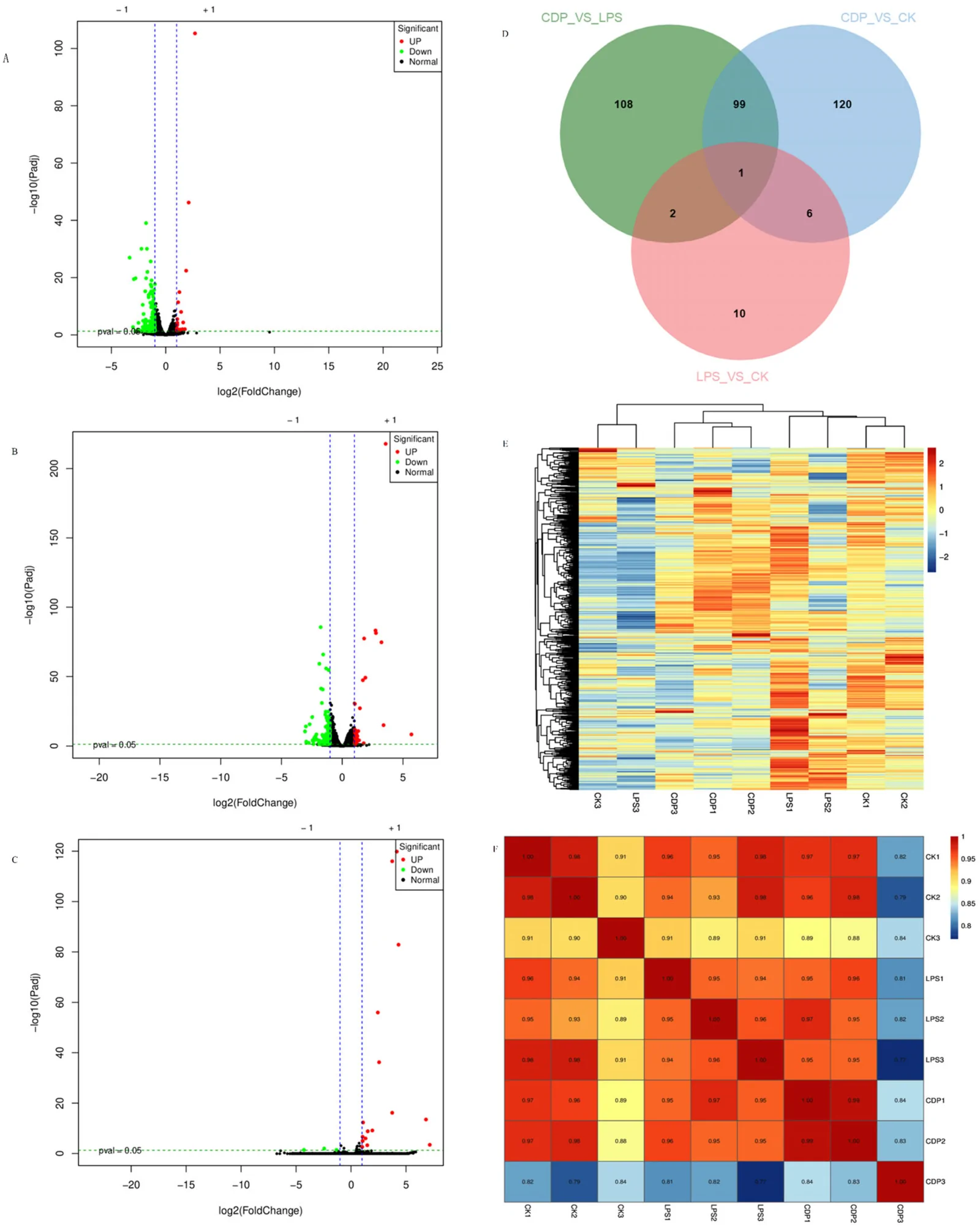
Transcriptome differences between the RAW 264.7 samples. Red dots and green dots represent genes differentially expressed in up-regulation and down-regulation, respectively. (**A**) CDP-VS-LPS; (**B**) CDP-VS-CK; (**C**) LPS-VS-CK; (**D**) using the Venn diagram for standard or unique DEGs; (**E**) heat map of the DEGs in different groups; (**F**) sample correlation heat map. Transcriptomic DEGs were identified using DESeq2 with FDR < 0.05 (adjusted *p*-value) and |log_2_FC| ≥ 1.

**Figure 7:**
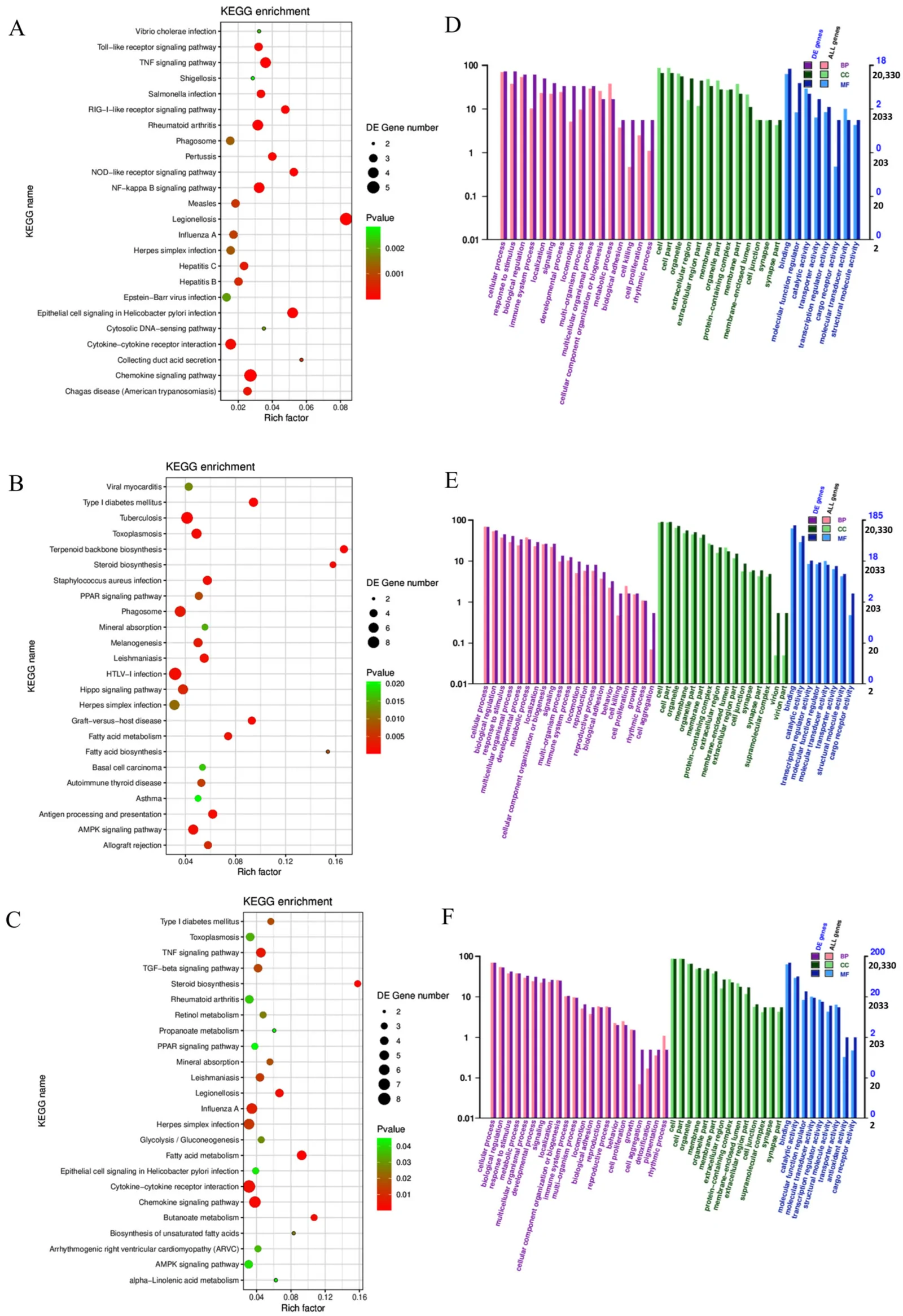
Enrichment analysis of differentially expressed genes (DEGs). (**A**–**C**) KEGG pathway enrichment analysis for the following comparisons: LPS vs. CK (**A**), CDP vs. LPS (**B**), and CDP vs. CK (**C**). (**D**–**F**) GO functional enrichment analysis for the following comparisons: LPS vs. CK (**D**), CDP vs. LPS (**E**), and CDP vs. CK (**F**). All enrichment results were filtered by FDR < 0.05, and generic terms were removed.

**Figure 8:**
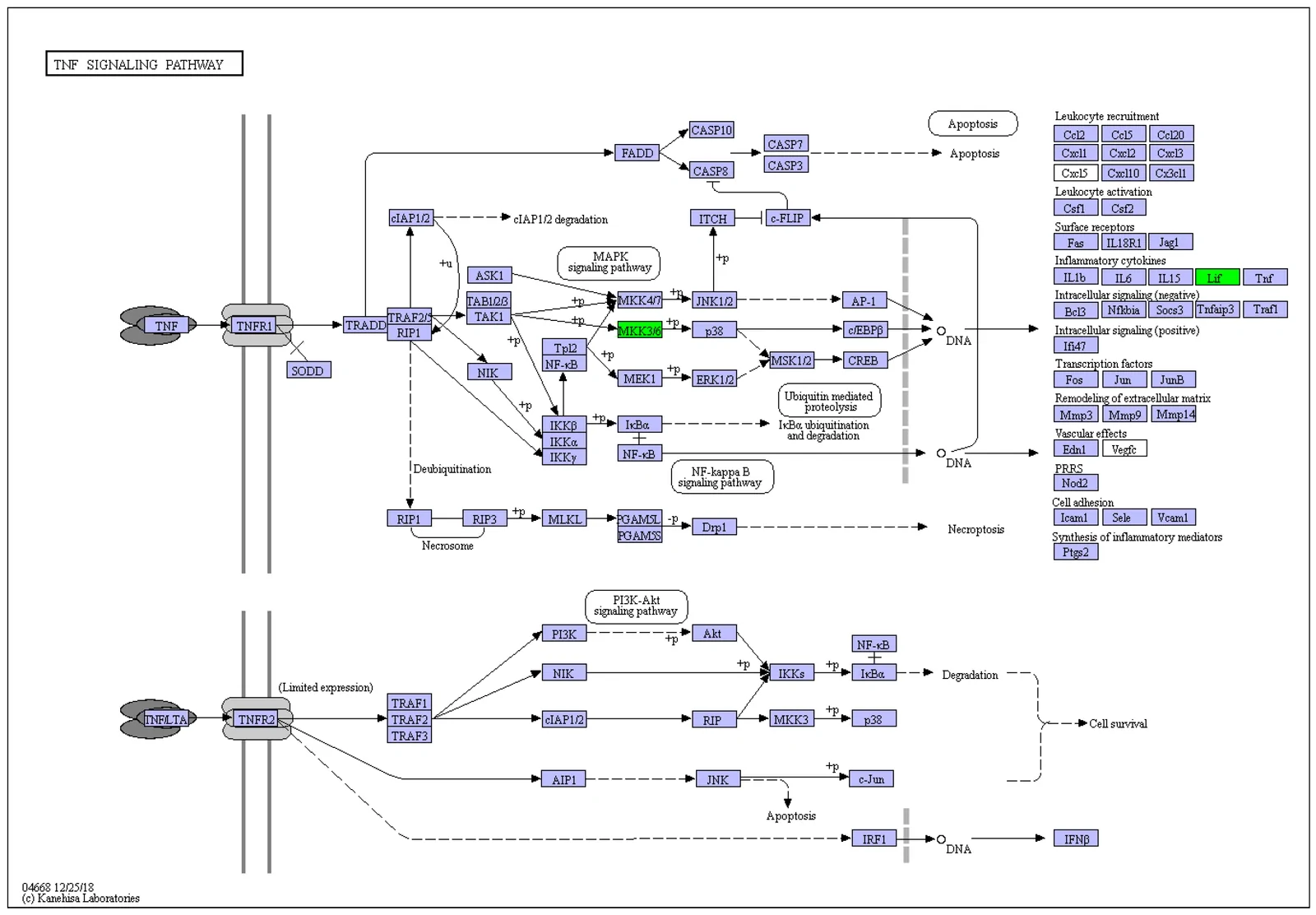
TNF signaling pathway with key differentially expressed genes highlighted. Green indicates down-regulated genes. Arrows represent activation or inhibition according to standard pathway conventions.

**Figure 9:**
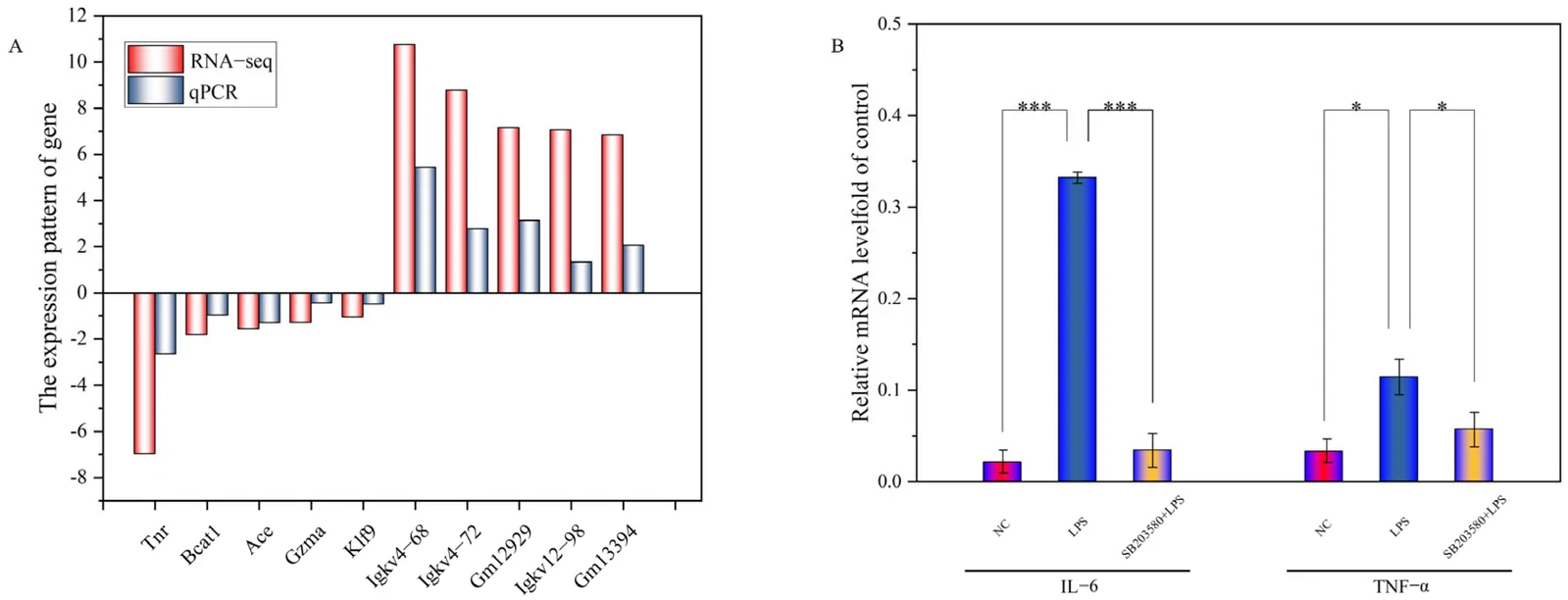
(**A**) qPCR validation of 10 selected DEGs (from CDP vs. LPS group) showing high concordance with RNA-seq. (**B**) Effect of p38 inhibitor SB203580 (10 μM, 1 h pretreatment) on LPS-induced IL-6 and TNF-α mRNA levels. * *p* < 0.05 and *** *p* < 0.001.

**Table 1:** Primer sequences used for gene expression.

Primer	GenBank Accession No	Product (bp)		Sequence (5′ → 3′)
IL6	NM_031168.1	308	F	CCACCCCAGGCAGACTACT
			R	CTGATTGAACCCAGATTGGAAGCAT
IL1	NM_008361.4	153	F	GTGTTCTGCATGAGCTTTGTG
			R	CCTCTTGGGGTAGACTTTGG
TNF-α	NM_013693.3	374	F	CGGTGGTGGGACTCGTATG
			R	GTTGACCTTGGTCTGGTAGG
TLR4	NM_021297.3	206	F	ACATCCCCATATCAACCAAGA
			R	GGATAGGGTTTCCCGTCAG
iNOS	NM_010927.4	499	F	ATGTCCGAAGCAAACATCAC
			R	TAATGTCCAGGAAGTAGGTG
β-actin	NM_007393.5	171	F	TGTTAGCTGCGTTACACCCT
			R	ACCTTCACCGTTCCAGTTTT
CIB2	NM_001164090.2	118	F	CCACCCCAGGCAGACTACT
			R	CTGATTGAACCCAGATTGGAAGCAT
HJV	NM_001081144.1	160	F	AATCTCCGGTGACCCTAAC
			R	AAAGAGCCCAGAAAGAAGTG
CIART	NM_028772.3	245	F	GGCTGAAGATGGGTCG
			R	TGGATAACTGTGGTGGC
LPIN1	NM_001291958.1	242	F	CCTCATCTGGCGGTTGC
			R	GGGCTGGACTCTTTCATCTTG
SPATC1L	NM_001033414.2	174	F	AAGGATGGTCGCATTGTTG
			R	CCGCAGCTTCTTCTCGTC
ABCA1	NM_013454.2	157	F	TTGGATGTTGGGTTGC
			R	TGCGGGAGTAAGGGTC
TMPRSS9	NM_001163705.1	197	F	CTGGAGGGCAAGGTGGAT
			R	GGGAGCAAGTGGGAGGACAT
MT2A	NM_008630.5	93	F	GCCATCCTTTGCTCAGCAGT
			R	CGTGCAGGATTCACCCGC
DNAH1	NM_001166310.1	200	F	GTGCTTCATCCACGGACTGT
			R	TCGATAGTGTTCCAGCACGG
TBCEL	NM_172618.4	219	F	TTGAACGCTATCGAGGAGCC
			R	GACGGATGGCAATCTGGCTA

**Table 2:** Summary of data quality tests.

Samples	Total Reads	Clean Reads	Percentage of Clean Reads	Clean Bases	GC Content	%>Q20	%>Q30
CDP1	42,200,752	42,192,892	99.98%	6,032,564,322	47.08%	97.92%	94.99%
CDP2	45,848,388	45,836,806	99.97%	6,543,265,602	47.08%	97.51%	94.14%
CDP3	87,130,414	87,090,938	99.95%	12,599,695,706	46.84%	98.34%	95.37%
CK1	62,007,054	61,988,814	99.97%	8,896,938,540	47.08%	97.83%	94.78%
CK2	41,312,286	41,304,086	99.98%	5,925,282,474	46.63%	97.85%	94.87%
CK3	41,564,224	41,556,268	99.98%	5,950,008,628	46.21%	97.69%	94.5%
LPS1	50,432,376	50,418,646	99.97%	7,237,589,214	47.63%	98.0%	95.09%
LPS2	58,757,702	58,740,962	99.97%	8,451,555,328	46.69%	97.82%	94.78%
LPS3	87,912,832	87,873,624	99.96%	12,596,668,932	45.39%	98.14%	94.75%

**Table 3:** Comparison reference genome statistics.

ID	Total Reads	Unmapped Reads (%)	Mapped Reads (%)	Secondary Alignments (%)	Unique Alignments (%)
CK1	61,988,814	3,051,869 (4.92)	58,936,945 (95.08)	1,772,999 (2.86)	57,163,946 (92.22)
CK2	41,304,086	1,852,652 (4.49)	39,451,434 (95.51)	1,158,440 (2.8)	38,292,994 (92.71)
CK3	41,556,268	1,953,265 (4.7)	39,603,003 (95.3)	1,083,789 (2.61)	38,519,214 (92.69)
LPS1	50,418,646	2,244,844 (4.45)	48,173,802 (95.55)	1,450,403 (2.88)	46,723,399 (92.67)
LPS2	58,740,962	2,797,173 (4.76)	55,943,789 (95.24)	1,651,672 (2.81)	54,292,117 (92.43)
LPS3	87,873,624	3,150,849 (3.59)	84,722,775 (96.41)	2,400,841 (2.73)	82,321,934 (93.68)
CDP1	42,192,892	2,018,732 (4.78)	40,174,160 (95.22)	1,311,953 (3.11)	38,862,207 (92.11)
CDP2	45,836,806	2,546,270 (5.56)	43,290,536 (94.44)	1,449,463 (3.16)	41,841,073 (91.28)
CDP3	87,090,938	2,514,764 (2.89)	84,576,174 (97.11)	2,716,530 (3.12)	81,859,644 (93.99)

## Data Availability

All data generated or analyzed during this study are included in this published article. All data and materials are the result of research and are available from the corresponding authors upon reasonable request, and are appropriately cited in the manuscript. All data analyzed or generated during this study are included in this published article.
